# Supplementary material for: Prevalence, nature and predictors of omitted medication doses in mental health hospitals: A multi-centre study
Source: PLoS One. 2020 Feb 6;15(2):e0228868. doi: 10.1371/journal.pone.0228868 (PMC7004323; doi:10.1371/journal.pone.0228868)
Supplement: Attachment — Submitted filename: RESPONSE TO REVIEWERS_RKeers_PLOSONE_v1.docx [file pone.0228868.s002.docx]

**RESPONSE TO REVIEWER COMMENTS**

**PONE-D-19-23592 ‘Prevalence, nature and predictors of omitted medication doses in mental health hospitals: a multi-centre study’**

**Editorial Comments:**

1.  Please ensure that your manuscript meets PLOS ONE's style requirements, including those for file naming. The PLOS ONE style templates can be found at

<http://www.journals.plos.org/plosone/s/file?id=wjVg/PLOSOne_formatting_sample_main_body.pdf> and <http://www.journals.plos.org/plosone/s/file?id=ba62/PLOSOne_formatting_sample_title_authors_affiliations.pdf>

*Acknowledged.*

2.  In ethics statement in the manuscript and in the online submission form, please provide additional information about the patient records used in your retrospective study. Specifically, please ensure that you have discussed whether all data were fully anonymized before you accessed them and/or whether the IRB or ethics committee waived the requirement for informed consent. If patients provided informed written consent to have data from their medical records used in research, please include this information.

*Acknowledged – we have now made clear in the ethics statement that data were fully pseudonymised before being sent to the research team for analysis and that individual patient consent was not required by the research ethics committee for this service evaluation study.*

**Reviewers' comments:**

Reviewer #1: The paper is very well written and the subject is also considerable

*Many thanks for these kind words and for reviewing our manuscript.*

Please also consider the following:
Please mention how 9 hospitals were selected? Did you get all the hospitals in the two centers?

*A total of 11 hospitals were covered by the 2 participating NHS trusts, with 1 being excluded as this focused on long-stay forensic wards rather than acute ward environments and another due to reasons of local resource capacity. Specialist wards such as long-stay forensics, intensive care and child and adolescent care were excluded in order to help ensure study findings were generalizable. The manuscript (page 6) and our ‘Strengths and Limitations’ section in the Discussion (page 20) has now been updated to reflect this point.*

How did you calculate the sample size needed for this study? How was the sampling done?

*As this study was an exploratory service evaluation to measure medicines administration quality we did not perform a sample size ‘power’ calculation. The number of data collection days (n=6) was chosen based on local capacity as each NHS trust provided the data collectors (we also refer to NHS team capacity in our ‘Strengths and Limitations’ section in the Discussion), and the days were scheduled to ensure data were collected over a 3 month period to better reflect usual working practices. All patients on each ward were included if eligible following screening. The manuscript has now been updated with this information (see page 7).*

Were patients also satisfied with the use of medical information?

*As per yours and additional editorial comments, we have added a statement to our ‘Ethical Approval’ section which states that individual patient consent was not required by the research ethics committee for this service evaluation study.*

Reviewer #2: The structure of the manuscript is well put together, however it has some defects that listed in below:
Results
- Line 179: “The rate of overall omissions including various subcategories is summarised in Table 1”. Why did you report total omitted doses including, ‘preventable’ omitted doses? If it is total, what about a column for ‘non-preventable’?

*Many thanks for reviewing our manuscript and for providing these valuable comments.
An important aim of our work was to present both the overall omitted dose rates, and the ‘preventable’ rates to the reader as findings concerning these ‘preventable’ omissions may help better focus local quality improvement efforts and future research in the field. However, we acknowledge that our focus on preventability of omissions could be clearer in our introduction and we have added text to achieve this (page 5) as well as in the discussion (page 17).

We present the overall numbers of ‘preventable’ and ‘non-preventable’ omissions in Table 3 so the reader can compare their relative burden, and by comparing the numerator figures across columns 1 and 2 in Table 1, the reader can also determine the rates for ‘non-preventable’ omissions. This helps to focus attention on ‘overall’ and ‘preventable’ omissions whilst avoiding making Table 1 larger and more difficult to process for the reader.*

- Please list out your exact findings related to predictors factors and then discuss about them. I cannot find them easily in the text, as a reader.

*Within the results section page 14, we have a section entitled ‘Predictors of omitted doses’ where we describe all the statistically significant findings reported in Tables 4 and 5. We have now expanded on this section to include all findings (whether significant or not) from the analysis.*

Ethical approval
- Further information is needed. For instance, any permission to access inpatient prescription charts?

*As per yours and additional editorial comments, we have added a statement to our ‘Ethical Approval’ section which states that individual patient consent was not required by the research ethics committee for this service evaluation study.*

Discussion
- Line 250: “We found evidence that particular patients may be experiencing more omitted doses”. What do you mean from "particular patients"?

*Many thanks for this comment. We have removed reference to ‘particular patients’ and have added additional details concerning the predictors for ‘preventable’ omissions in this paragraph.*

- Discussion more about implications of findings in clinical practice.

*Based on your earlier helpful comments we have added some detail to the Discussion concerning the implications for preventable omissions. We have also now reviewed the Discussion and added lines of text across sections to help better link the findings to clinical practice, including ‘preventable’ omissions occurring at weekends and addressing patient refusals as a driving factor behind non-preventable omissions.*
